# igraph 1.0 enables fast and robust network analysis across programming languages

**DOI:** 10.1371/journal.pone.0355108

**Published:** 2026-08-04

**Authors:** Michael Antonov, Gábor Csárdi, Szabolcs Horvát, Kirill Müller, Tamás Nepusz, Daniel Noom, Maëlle Salmon, Vincent Traag, Brooke Foucault Welles, Fabio Zanini

**Affiliations:** 1 cynkra GmbH., Zürich, Switzerland; 2 Posit Software, PBC, Boston, Massachusetts, United States of America; 3 Reykjavik University, Reykjavík, Iceland; 4 Center for Systems Biology Dresden, Dresden, Germany; 5 Sixdegrees Ltd, Budapest, Hungary; 6 jitjit, Amsterdam, Netherlands; 7 Centre for Science and Technology Studies, Leiden University, Leiden, Zuid-Holland, Netherlands; 8 Northeastern University, Boston, Massachusett, United States of America; 9 School of Clinical Medicine, UNSW Sydney, Sydney, NSW, Australia; 10 Evolution & Ecology Research Centre, UNSW Sydney, Sydney, NSW, Australia; Ahmedabad University, INDIA

## Abstract

Networks or graphs are widely used across the sciences to represent relationships of many kinds. The igraph (https://igraph.org) software library supports graph construction, analysis, and visualisation, combining fast and robust performance with a low entry barrier. igraph pairs a fast core written in C with beginner-friendly interfaces in Python, R, and Mathematica. After twenty years of development, igraph 1.0 has been released, enabling public access to a robust and stable network analysis with over a million monthly downloads. Thanks to its cross-language design, igraph delivers both speed and flexibility: it can handle billions of edges, supports interactive plotting, integrates with notebooks, facilitates conversions to and from other network libraries, includes a rich library of graph layout and community detection algorithms, and has a detailed documentation including non-English translations. Modern testing features such as continuous integration, address sanitizers, stricter typing, and memory-managed vectors have also increased robustness. Hundreds of bug reports have been fixed and a community forum has been opened to connect users and developers. Specific effort has been made to broaden use and community participation by women, non-binary people, and other demographic groups typically underrepresented in open source software.

## Introduction

Networks or graphs are widely used across multiple scientific disciplines. In mathematics, graphs have been studied for centuries from a theoretical perspective and are still a topic of considerable interest. Random graphs, initially defined by Erdős and Rényi [1], and more recently scale-free stochastic networks, have sparked a new wave of interest aimed at understanding the analytical properties of graphs generated according to statistical rules, with real world applications in engineering, finance, and biology [2]. In biology and medicine, the genomics revolution driven by next-generation sequencing has brought graphs into the spotlight for a wide array of applications, from De Bruijn graphs for genome assembly [3] to cell similarity networks in cell atlases [4]. Social network analysis has a long history, dating back to Moreno’s sociometry [5], and includes the widespread study of social systems, ranging from friendships to organizations and social movements [6–8]. The advent of large online behavioural data opened up many new possibilities, helping give rise to computational social science [9], in which networks play a pivotal role.

Open source computational tools are of central importance in the investigation of graphs. In the case of random graphs, computers enable the simulation of thousands of such graphs at scale and allow empirical computations of their statistical properties. Real-world networks such as protein-protein interactions, single-cell transcriptomics, citation networks or online social networks have thousands or even millions of vertices. High-performance and efficient software libraries are therefore absolutely necessary for their analysis and manipulation. A few general-purpose graph analysis libraries are widely used. NetworkX is a popular library in the Python community [10] that is written in pure Python. Graph-tool is also used by many researchers for its faster speed and its focus on stochastic networks [11]. NetworKit is a fast Python/C++ library that includes parallelized algorithms [12] and rustworkx combines a Python, NetworkX-like API with a Rust core library [13]. StatNet is an R library geared towards dynamic networks and statistical inference that is popular among social scientists [14]. Boost Graph, which implements about a dozen core functions [15], and SNAP, which includes about 100 functions and was last updated in 2020 [16], are available in C++. In addition to graph analysis, graph visualisation is also essential across multiple scientific disciplines. Cytoscape is a popular choice for advanced graph visualization [17], while D3.js can be used from JavaScript to embed networks in a web page [18]. Gephi is a popular network analysis tool with a central role for graph visualization [19], and both NetworkX and graph-tool can produce relatively simple visualisations from Python. Despite this vibrant software ecosystem, there is a strong demand for a general-purpose graph analysis library that is fast, consistent across programming languages, cross-platform, easy to install, and well documented. igraph meets these needs by providing efficient implementations of widely used algorithms, many of which are not available in any other library, including for determining shortest paths [20], vertex centrality [21,22], cycle bases [23], motif finding [24], clique finding [25], community detection [26], and graph layout [27].

## Materials and methods

### Performance and comparison tests

**Fig 1:**
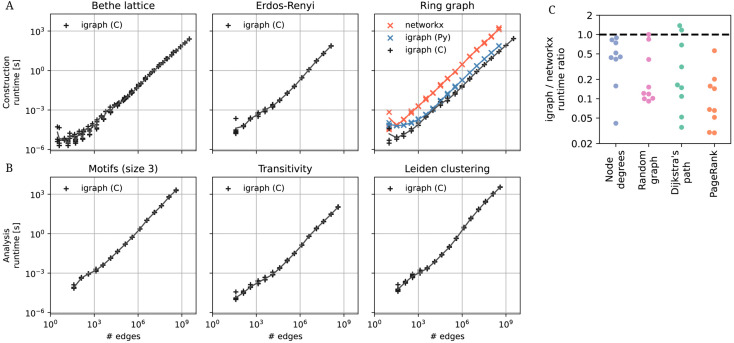
Runtime scalability of igraph. (**A-B**) Runtimes to construct (**A**) and analyse (**B**) graphs of varying sizes. Top row: Construction runtime of an undirected Bethe lattice or regular tree, an Erdős–Rényi or random graph and a directed ring graph using the igraph C core directly and, for the latter, the Python igraph interface and the networkx library. Bottom row: Analysis runtime – excluding graph construction time – for size-3 motif search, transitivity, and Leiden clustering using the C core. Each graph size was tested 10 times (crosses). Solid lines: averages for each graph size. (**C**) ratios of average runtimes between igraph (python interface) and networkx for four example algorithms across graph sizes (1-30,000 nodes).

Scalability tests for Fig 1 were performed on a tower server with 512 GB of RAM and using a single Intel Xeon Platinum 8160T CPU at 2.10GHz. Comparisons against scikit-network (Supplementary Figures) were performed on a laptop with 32GB RAM and Intel Core Ultra 7 265H cpus. All tests were run 10 times with memory deallocation/garbage collection between runs and libraries. Library versions: igraph 1.0.0, networkx 3.6.1, scikit-network 0.33.0 and graph-tool 2.98 (Supplementary Figures).

### Graph layouts

To demonstrate the graph layout capabilities of *igraph*, we constructed graphs of increasing size. Each graph is built as a set of 10 groups of vertices that are tightly connected internally and with random distance between groups. The 2D Fruchterman-Reingold algorithm (force variant for graphs with less than 500 nodes, energy variant for larger graphs) and the UMAP algorithm [27] were run on the same graph for sizes up to 1,000 vertices. For the 100,000-node graph, only UMAP was run for performance reasons. Notice that UMAP is not designed to work on very small (e.g., 30-node) graphs. All plots use the new matplotlib backend for igraph.

### Package statistics

Download statistics for PyPI were computed using PyPI stats (https://pypistats.org/). Download statistics for R were computed using cranlogs.app (https://github.com/r-hub/cranlogs.app). Counts of edited lines, forks, and stars were computed using GitHub’s online interface. The number of functions in Python were estimated using the inspect standard module. The number of functions in R were estimated using lsf.str(“package:igraph”).

## Results

### Growing a large userbase across four programming languages

Since the initial publication in 2006 [28], igraph has grown in multiple areas, including the C core, extensions in Python, R, and Mathematica [29]. The codebase has had around 3 million lines of code edited across approximately 10,000 versioning commits. As of June 2026, the four main code repositories, one for each programming language, have been forked approximately 800 times and starred 3,000 times. During that month, the Python and R packages were downloaded around 2.1 million and 600 thousand times respectively, which is a conservative estimate given that tracking downloads is challenging.

### Scaling network analysis to billions of edges through a fast C core

igraph is based on a low-level core codebase in C, on top of which higher-level language interfaces (Python, R, Mathematica) are built to leverage high-level languages’ conciseness without loss of speed (see S1 Fig for a cross-language example). The C core includes approximately 1,100 user-facing functions. Since 2006, three thousand files have been edited and 154 individual features added to the C core (S1 Table). The build system has been designed to enable easy cross-platform installation from source in Linux, macOS and Windows. Precompiled packages are also available at the system level (e.g., Ubuntu, Debian, and Alpine Linux, Homebrew and MacPorts for macOS, and vcpkg for Windows) and the language-specific extensions are available on PyPI for Python, the comprehensive R network (CRAN) for R, and as a Paclet for Mathematica.

To reach the largest audience, igraph relies on a compile-time configurable integer size. This enables support of users on 32-bit embedded devices but also 64-bit desktops, laptops, and servers, enabling analysis of networks with billions of vertices and edges. As an example, constructing an undirected Bethe lattice with 3.2 billion edges took 4 minutes on a server using a single CPU core (Fig 1A). Similar results were obtained when constructing a random graph with 400 million edges and a directed ring graph with 3.3 billion edges (Fig 1A). In addition to graph construction routines, several graph analysis routines were also assessed for runtime (Fig 1B) performance and memory usage (S2 Fig). Comparison of direct usage of the C API versus the igraph Python interface showed significant overhead for small graphs, reducing the performance of igraph’s Python code to networkx’s speed, but very little overhead for large graphs, where igraph’s execution is approximately 10 times faster than networkx. igraph’s R interface is more limited in terms of network size due to R’s internal usage of double precision floating point numbers to represent integers. To further assess igraph’s speed against networkx, we computed runtime ratios for four algorithms across a wide range of graph sizes (1–30 thousand nodes) and found that igraph was faster in essentially all cases, usually by approximately one order of magnitude (Fig 1C and S3 Fig). Comparisons with graph-tool and scikit-network were also performed (S4 Fig).

igraph includes hundreds of functions for graph construction, I/O, structural analysis, paths or traversals, community detection, and layouts. Community detection includes, among others, Leiden [26], multilevel (or Louvain) [30] and fluid communities [31]. Graph layout algorithms include Fruchterman-Reingold, multidimensional scaling [32], the Large Graph Layout [33], Davidson-Harel [34], the GEM layout [35], Kamada-Kawai, and an implementation of Uniform Manifold Approximation and Projection (UMAP) [27] that does not depend on cross-language packages such as numba and llvmlite.

### Broad accessibility to R, Python, and Mathematica users

**Fig 2:**
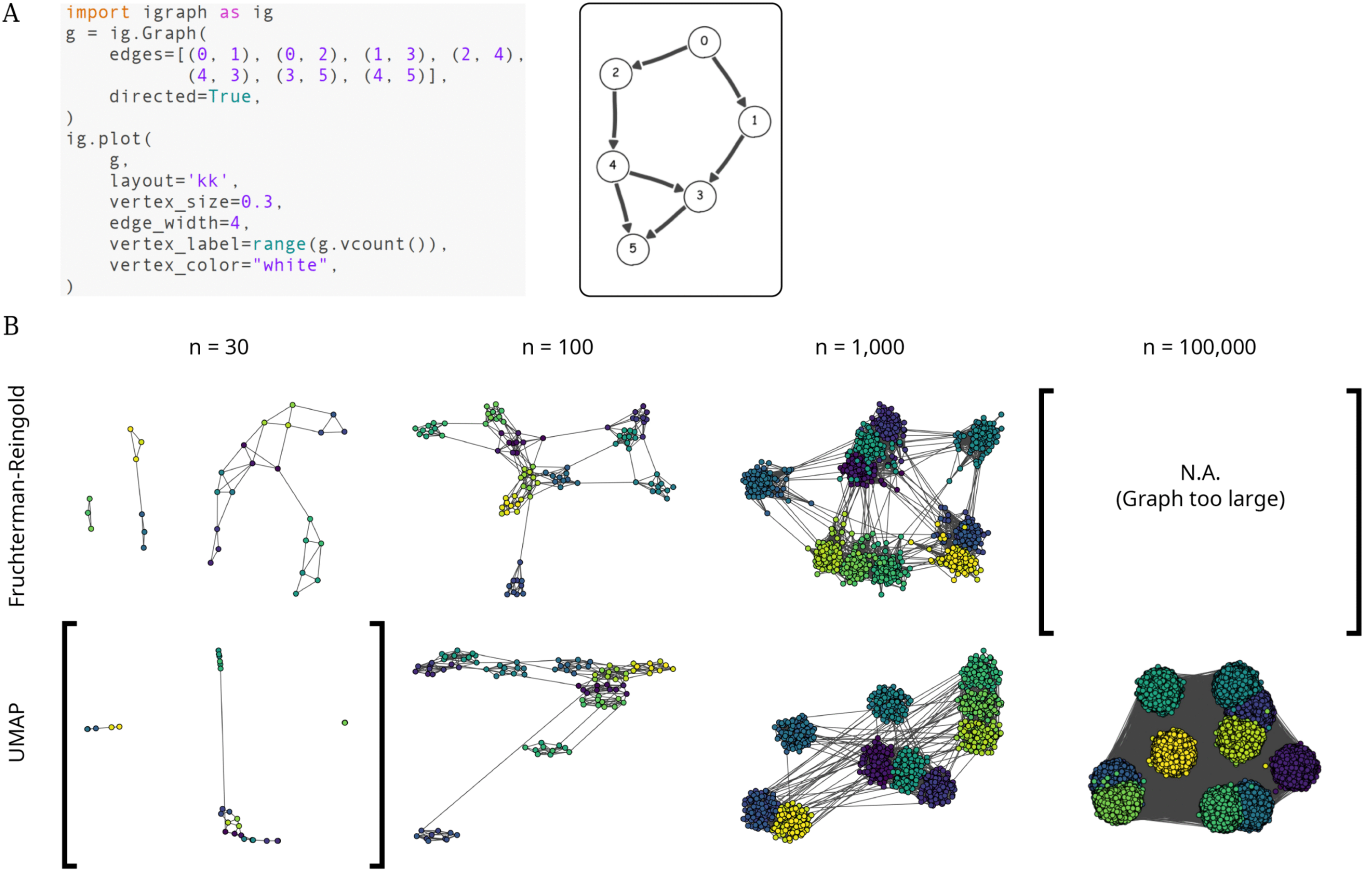
igraph enables simple and interactive graph visualisation. (**A**) Example code to plot a simple graph with topological sorting. (**B**) Examples of graph layouts in igraph. The spring-based Fruchterman-Reingold layout and the Uniform Manifold Approximation and Projection (UMAP) layout algorithms [27] for graphs of increasing size. Plots within brackets are outside of the typical range of applicability of the respective algorithm.

**Fig 3:**
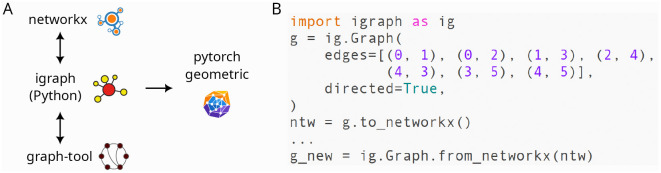
Linking network libraries together. **(A)** igraph’s Python interface can exchange in-memory graphs with networkx, [10] graph-tool [11], and pytorch geometric [40]. **(B)** Example of a network conversion from igraph to networkx and back using the new import/export functions, which preserve attributes as well.

The Python interface, which includes around 600 functions, is compatible with Jupyter notebooks, a browser-based environment for scientific computing and rapid prototyping that supports both R and Python and is widely used in education, research, and industry [36]. The Python visualisation layer has also been redesigned to support not only the existing Cairo backend but also matplotlib [37] and plotly [38], which enable interactive plots (e.g., zoom and pan) and animations (Fig 2). Furthermore, igraph’s Python interface supports in-memory import/export of graphs to and from networkx [10] and graph-tool [11], two popular network analysis libraries (Fig 3A). Conversions preserve graph, vertex, and edge attributes, making it easy to convert an igraph graph into a networkx object, call a specific function on it, then convert back to igraph for further analysis (Fig 3B). Moreover, conversions are fast (S5 Fig): generating a graph in *igraph* and then converting it to a library of choice can sometimes be faster than using native constructor routines. Compatibility with pandas dataframes has been added as well [39]. Finally, we aimed to facilitate the use of igraph as a data preparation step for graph-based machine learning by adding a function that exports networks from igraph to pytorch geometric [40] (Fig 3A).

The R interface of igraph supports R 4.0 and later and comprises approximately 750 user functions. As of June 2026, on CRAN, the main code repository for the R language, igraph is imported by 713 packages: igraph has even been used to compute CRAN’s own dependency graph [41]. Most R functions are wrappers around fast C functions, however the user interface and the structure of the package are idiomatic to R to lower the entry barrier for contributors.

A Mathematica interface is available at http://szhorvat.net/mathematica/IGraphM. Unlike the C, R, and Python interfaces, it is mainly designed to extend the network analysis capabilities of the Mathematica standard library rather than to replace them. For instance, the Mathematica interface exposes useful community detection, layout, motif finding, and random graph generation algorithms that are otherwise not available. It also implements a dedicated interactive editor to construct graphs visually, which is especially appealing for prototyping or brainstorming. Details of the Mathematica interface were described in a recent publication [29].

### Documentation and testing

igraph has an extensive and robust testing framework. Continuous integration across multiple architectures (64-bit Linux for Intel and AMD CPUs, macOS for 64-bit Intel and the Apple Silicon M-series CPUs, and Windows), including usage of address sanitizers, ensures that the codebase is not just well tested but that new contributions are thoroughly vetted as well. Test coverage for the C core is 85% excluding vendored dependencies which are already tested separately. In the Python interface, visualization functions are tested by pixel-by-pixel matching of the resulting images, as in matplotlib [37].

igraph’s documentation includes an R package vignette and documentation website using pkgdown [42] designed to help new users. The Python interface is provided through readthedocs.org and includes a gallery of examples available as scripts or Jupyter notebooks [43]. Translations for both the R and Python interface tutorials into Spanish are available, and other translations (e.g., Mandarin) are planned.

### Open science development towards a diverse community

**Fig 4:**
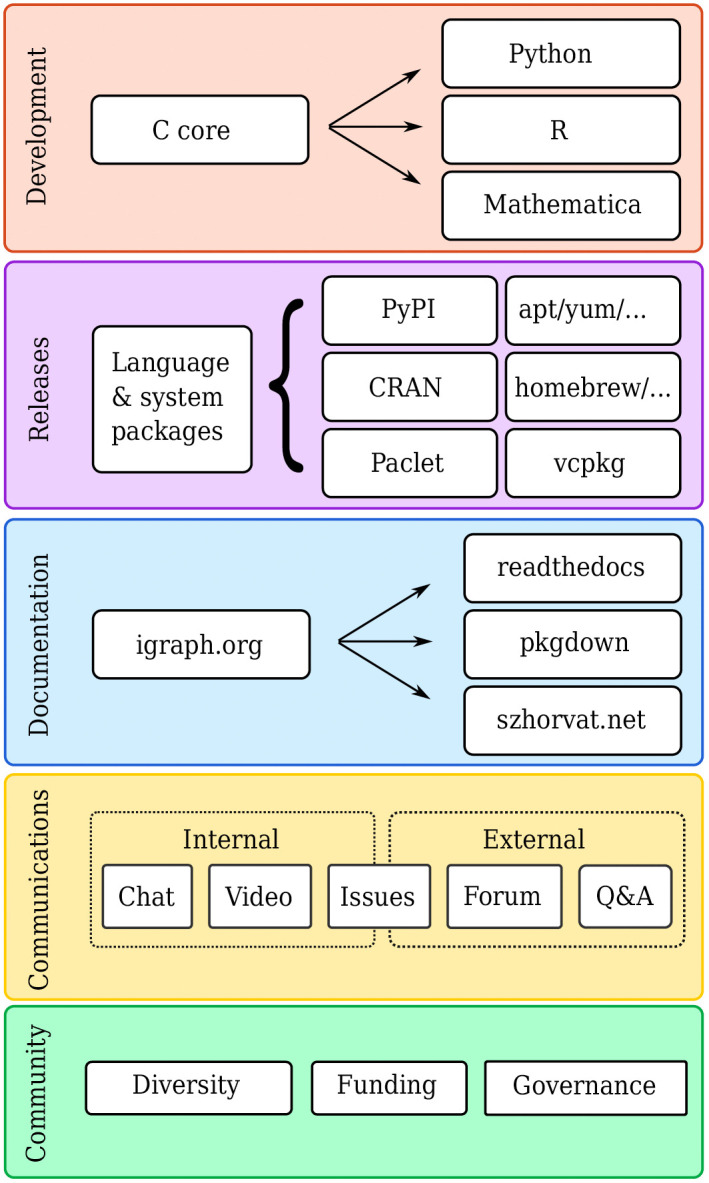
Schematic of igraph’s open source/open science infrastructure. From the top: development, releases, documentation, internal and external communication channels, and initiatives.

We continue aiming to grow the contributor and user bases (Fig 4) via transparent development practices. Repository issues are the agora where aspiring contributors interact with the core development team on specific bugs, feature requests, or enhancement proposals. Automatic testing of new contributions via continuous integration is configured openly, with results accessible by anyone. Releases are triggered for the popular dissemination platforms for each language (e.g., PyPI for Python, CRAN for R) and relationships with downstream package managers are actively maintained. The igraph documentation is deployed using language-specific conventions, which feels familiar even to new users. In addition to internal communication on development via chat rooms and regular video calls, igraph has a public user forum at https://igraph.discourse.group/ that is regularly visited by both developers and users. The developers also check in on online Q&A platforms such as Stack Overflow to provide answers.

The recent collaboration with Women in Network Science (WiNS) aims to extend our user and contributor base to a more diverse group of individuals, especially from women, non-binary people, and people from other groups that are traditionally underrepresented in open source. A team led by Foucault Welles ran a workshop with Women in Network Science that trained 25 women to use *igraph* for network analysis and visualization. Most participants had been unaware of *igraph* before the workshop, but successfully used *igraph* for network analysis and network visualization projects during the workshop. All expressed interest in continuing to use *igraph* in their scientific work, suggesting that targeted workshops are an effective strategy for onboarding new diverse users. In addition, the team conducted interviews with 19 open-source community managers lasting 60–90 minutes each on strategies for increasing diversity, inclusion, and belonging in open-source communities [44]. Briefly, these interviews suggested that the work of recruiting and onboarding new users and developers was universally recognized as important but often sidelined in favor of more technical tasks. Projects that successfully maintain diverse user and contributor bases had transparent governance structures with leaders specifically assigned to community maintenance tasks such as onboarding new users and giving constructive feedback to new contributors [44]. Based on these results, *igraph* has recently transitioned to a transparent governance structure with a Steering Committee that includes members responsible for strengthening the relationship between core developers and the broader user and developer community.

## Discussion

The computational analysis of graphs and networks has become a foundational element across scientific disciplines including mathematics, physics, engineering, biology and medicine, and the social sciences. It is also essential in industry settings, such as graph-based machine learning. A general-purpose, open source network analysis software library is essential for academia, start-up companies, non-profit organisations, and for other small teams and individuals. Over the last decade, igraph has morphed from a remarkable hobby project of two developers into an essential tool across disciplines and industries.

igraph’s scalability to billions of edges on consumer hardware is poised to simplify analytics on important graphs that are challenging due to their sheer size. Huge networks, which include a Twitter network of 40 million vertices and 1.5 billion edges [45] and a citation network with more than 240 million vertices and almost 2 billion edges [46], have become increasingly common in recent years. Beyond current data capabilities, igraph is ready for future analyses of global networks, for instance in epidemiology (8 billion people) and on social media (e.g., Facebook, 3 billion users). In turn, the results of such large computations can inform research and decision making in areas such as public health and online content moderation.

Interfaces in high-level languages have seen widespread use across the sciences due to their relatively flat learning curve and affinity for exploratory data science. In particular, systems biomedicine is a growing field where networks are of paramount importance. Cell-cell similarity graphs in single cell omics [47], gene regulatory networks [48] and protein-protein interaction networks [49] are fundamental tools to rationalize the complexity of biological systems such as the human body. The Python and R interfaces of igraph are widely used in some of these areas and the improvements implemented in the last years are designed to create a solid basis for further expansion in these areas.

One of the fastest growing areas of application of graphs is machine learning (ML), including graph-based sparse matrices [50], kernels [51], and graph neural networks (GNNs) [52]. Although igraph does not focus on ML applications, its efficient and generic design is amenable to tighter integration workflows with ML libraries. For instance, GraKeL is a Python graph kernel library that instantiates graphs from adjacency lists or dictionaries [53], both of which can be already obtained from the Python igraph interface. Extending igraph to export to GraKeL or, vice versa, extending GraKeL to import from igraph would be a straightforward task. A similar GNN-oriented export function for pytorch geometric required only 15 lines of code.

igraph’s design incurs some limitations. The current internal data structure is based on numbered arrays of vertices and edges, making it particularly efficient for static graph analysis but less performant for dynamic networks, i.e., networks in which vertices and edges are frequently added or removed. Although this is only noticeable on very large networks or for long simulations, future work is planned to provide a swappable core data structure that adapts to distinct use scenarios.

Another challenge of current igraph development is the entry barrier towards new code contributors. Although igraph’s codebase is vast and spans multiple languages, we are committed to creating a welcoming environment for new contributors. In particular, the joint operation with WINS is an exciting opportunity to include new contributors who identify with groups that have been historically underrepresented in igraph’s development team, such as women and non-binary people. As igraph’s coding standards continue to improve, it will be especially important to “walk a few steps” towards new community members in a context of mutual respect and positive encouragement. A new structure of formal governance, together with tighter interoperability with networkx and other packages, are key areas that will help increase accessibility for all types of users and developers.

## Supporting information

S1 Fig Example of igraph usage across programming languages. The code computes edge betweenness for a simple, manually constructed graph.
(PDF)

S2 Fig Memory usage of igraph across graph sizes. Transitivity and Leiden clustering were computed for random graphs and assessed by malloc_stats() using igraph’s C core as in Figure 1B. Tests run on a tower server with 512 GB RAM and Intel Xeon cpus.
(PDF)

S3 Fig Runtimes of igraph (Python) versus networkx for four algorithms across graph sizes. Tests run on a tower server with 512 GB RAM and Intel Xeon cpus. Tests run on networkx 3.6.1 and igraph 1.0.0.
(PDF)

S4 Fig Runtimes of igraph (Python) versus graph-tool and scikit-network across graph sizes. (A) Leiden clustering of the Zachary karate club network (left) and of random graphs of various sizes (right) for scikit-network and igraph. Runtimes refer to community detection only, not graph construction. Comparisons performed on a laptop with 32GB RAM and Intel(R) Core(TM) Ultra 7 265H cpus, using scikit-network 0.33.0 and igraph 1.0.0. (B) Global transitivity for the Zachary karate club network (left) and random graphs of various sizes (m = 4 * n, with m = number of edges, n = number of nodes) for graph-tool and igraph. For the left and centre panels, runtimes include only the transitivity computation, not the graph construction. The right panel shows graph construction times for the random graphs of the middle panel. Total wall time is the sum of the analysis and construction runtime. Note that igraph is much faster than graph-tool to construct this particular type of graph, but graph-tool is faster (2-4x) for transitivity computations specifically of large graphs. Comparisons performed on a tower server with 512 GB RAM and Intel Xeon cpus, using graph-tool 2.98 and igraph 1.0.0.
(PDF)

S5 Fig Runtimes for in-memory conversions of networks between igraph, networkx, and graph-tool. Each comparison was measured 10 times on a tower server with 512 GB RAM and Intel Xeon cpus using networkx 3.6.1, graph-tool 2.98, and igraph 1.0.0. Conversions from other libraries to igraph with large networks were limited by the runtime required to construct the network (in the external library).
(PDF)

S1 Table Tabular history of features in *igraph.* (XLSX)
