# Supplementary material for: igraph 1.0 enables fast and robust network analysis across programming languages
Source: PLoS One. 2026 Aug 4;21(8):e0355108. doi: 10.1371/journal.pone.0355108 (PMC13436765; doi:10.1371/journal.pone.0355108)
Supplement: Supplementary file 1 [file pone.0355108.s001.pdf]

# C core

```c
#include <igraph.h>

int main() {
    igraph_t graph;
    igraph_vector_t ebs;
    igraph_vector_int_t edges;
    igraph_es_t eids;

    /* Setup library */
    igraph_setup();

    igraph_vector_int_init(&edges, 4);
    VECTOR(edges)[0] = 1;
    VECTOR(edges)[1] = 2;
    VECTOR(edges)[2] = 1;
    VECTOR(edges)[3] = 5;

    /* Create the graph */
    igraph_create(
        &graph,
        &edges,
        10,
        IGRAPH_UNDIRECTED
    );

    /* Compute edge betweenness */
    eids = igraph_ess_range(0, 2);
    igraph_vector_init(&ebs, 0);
    igraph_edge_betweenness(
        &graph,
        NULL,
        &ebs,
        eids,
        IGRAPH_UNDIRECTED,
        false
    );

    /* Print result */
    for (int i=0; i < 2; i++) {
        printf("%f ", VECTOR(ebs)[i]);
    }
    printf("\n");

    igraph_vector_int_destroy(&edges);
    igraph_vector_destroy(&ebs);
    return 0;
}
```

# Python

```python
# Import igraph library
import igraph as ig

# Construct a graph
g = ig.Graph(
    edges=[(1, 2), (1, 5)],
    n=10,
    directed=False,
)

# Compute edge betweenness
ebs = g.edge_betweenness()

# Print result
print(ebs)
```

# R

```r
# Load igraph library
library("igraph")

# Construct a graph
g <- make_graph(
  edges = c(1, 2, 1, 5),
  n = 10,
  directed = FALSE
)

# Compute edge betweenness
ebs <- edge_betweenness(g)

# Print result
print(ebs)
```
