# Supplementary material for: igraph 1.0 enables fast and robust network analysis across programming languages
Source: PLoS One. 2026 Aug 4;21(8):e0355108. doi: 10.1371/journal.pone.0355108 (PMC13436765; doi:10.1371/journal.pone.0355108)
Supplement: Supplementary file 2 [file pone.0355108.s002.pdf]

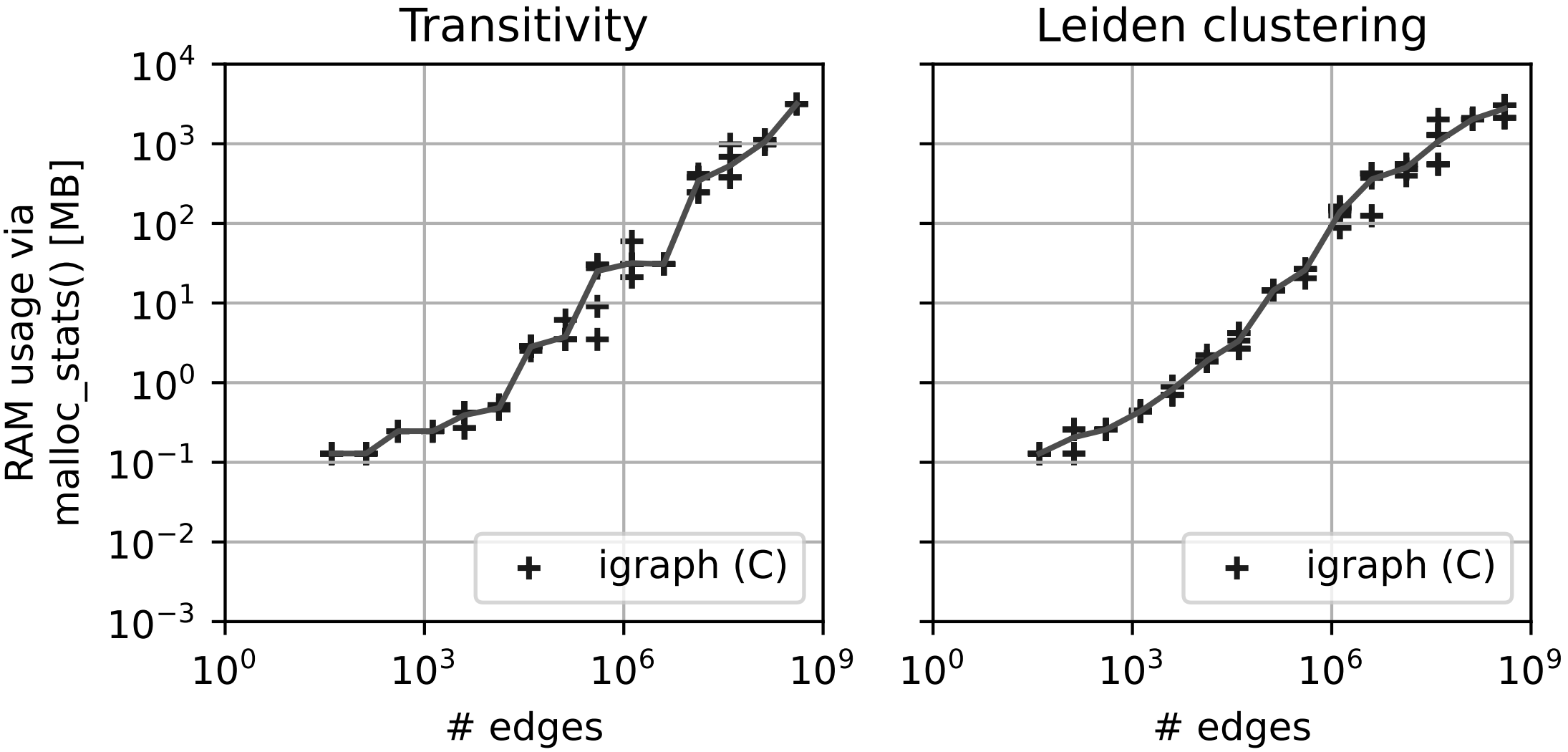
Transitivity
Leiden clustering
RAM usage via malloc_stats() [MB]
edges
igraph (C)
10^{-3}
10^{-2}
10^{-1}
10^{0}
10^{1}
10^{2}
10^{3}
10^{4}
10^{0}
10^{3}
10^{6}
10^{9}
