# Supplementary material for: igraph 1.0 enables fast and robust network analysis across programming languages
Source: PLoS One. 2026 Aug 4;21(8):e0355108. doi: 10.1371/journal.pone.0355108 (PMC13436765; doi:10.1371/journal.pone.0355108)
Supplement: Supplementary file 3 [file pone.0355108.s003.pdf]

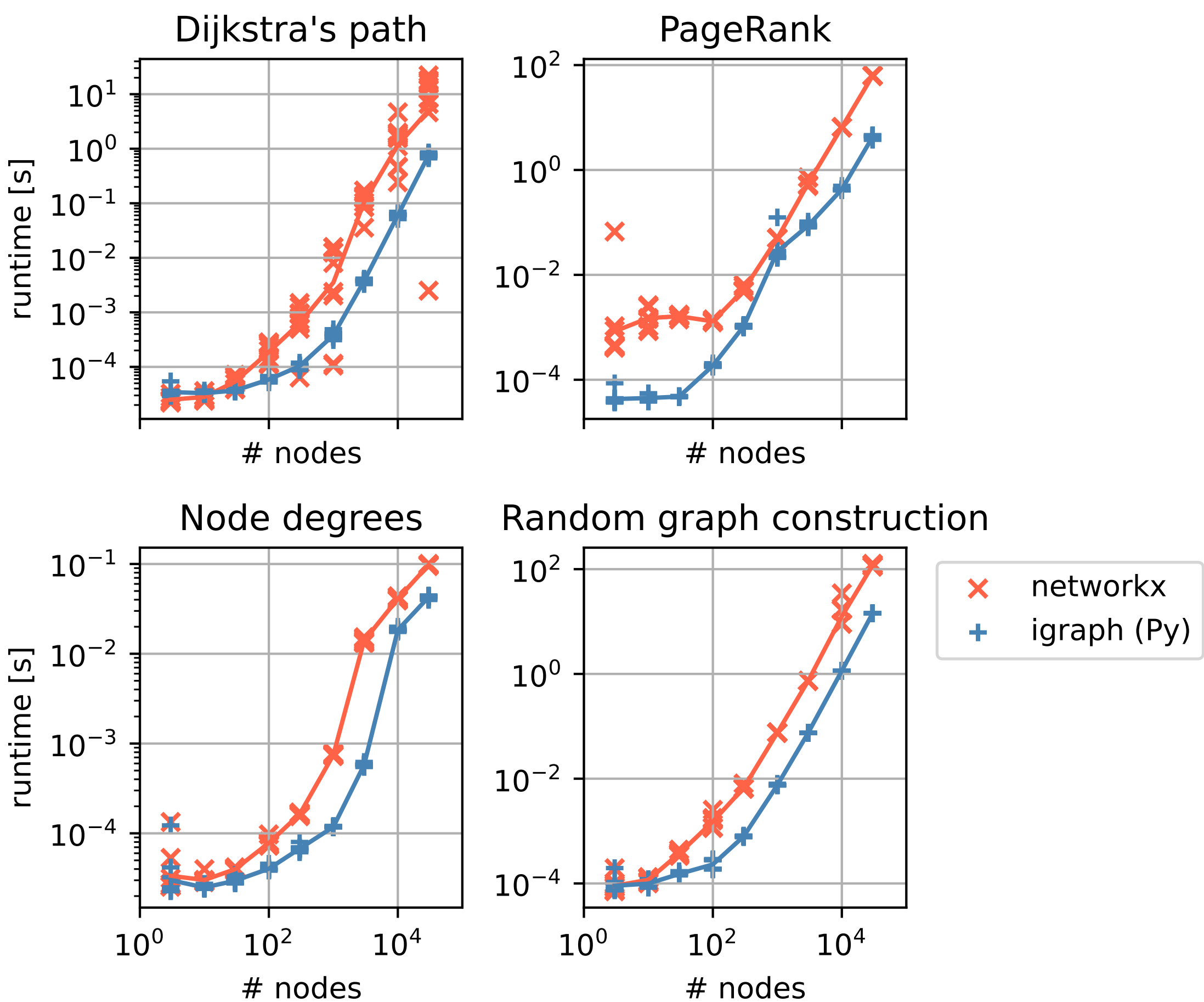

Dijkstra's path
PageRank
Node degrees
Random graph construction
runtime [s]
nodes
10^{1}
10^{0}
10^{-1}
10^{-2}
10^{-3}
10^{-4}
10^{2}
10^{0}
10^{4}
networkx
igraph (Py)
