# Supplementary material for: igraph 1.0 enables fast and robust network analysis across programming languages
Source: PLoS One. 2026 Aug 4;21(8):e0355108. doi: 10.1371/journal.pone.0355108 (PMC13436765; doi:10.1371/journal.pone.0355108)
Supplement: Supplementary file 4 [file pone.0355108.s004.pdf]

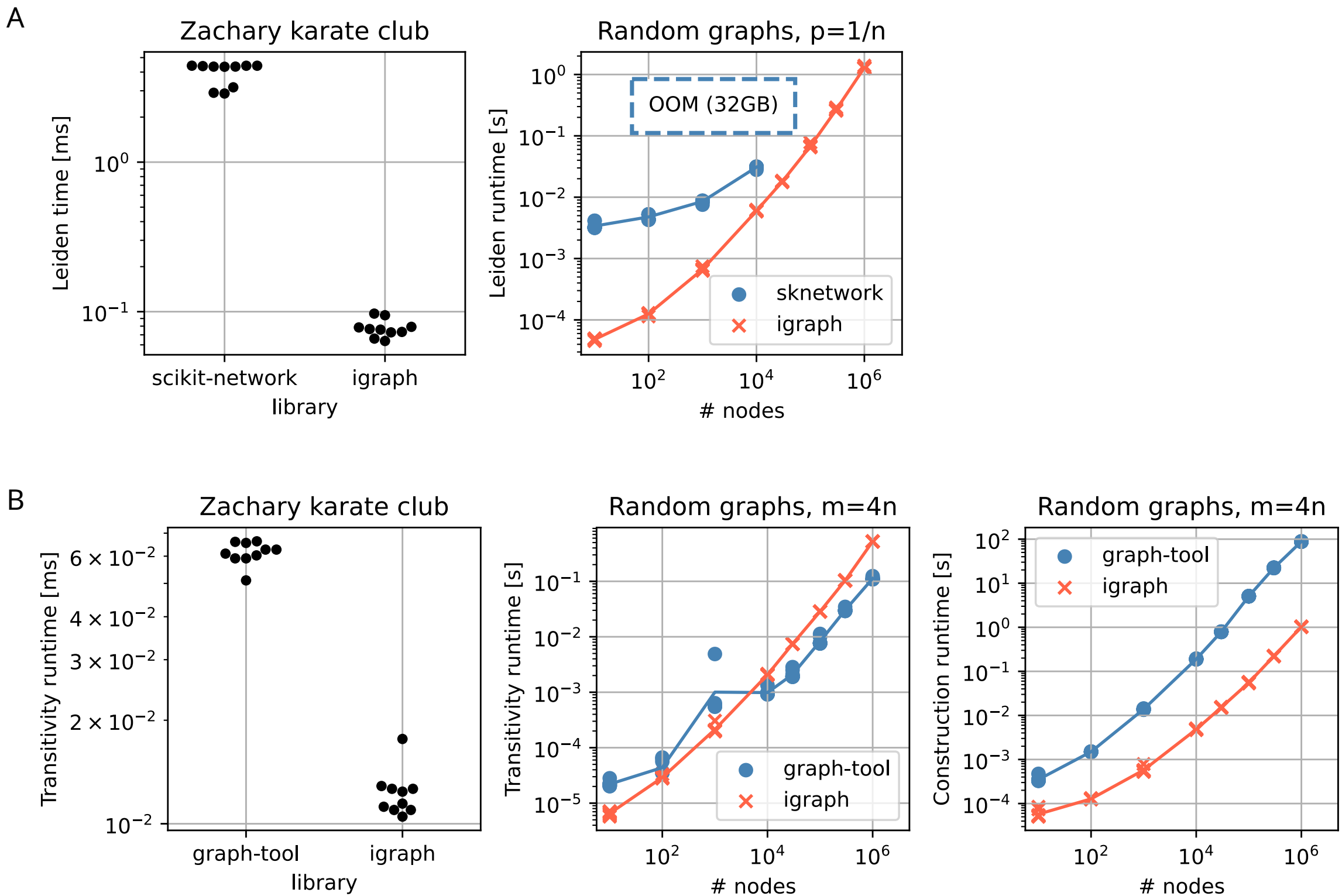
A
Zachary karate club
Leiden time [ms]
10^0
10^{-1}
scikit-network
igraph
library
Random graphs, p=1/n
Leiden runtime [s]
10^0
10^{-1}
10^{-2}
10^{-3}
10^{-4}
OOM (32GB)
sknetwork
igraph
10^2
10^4
10^6
nodes
B
Zachary karate club
Transitivity runtime [ms]
6×10^{-2}
4×10^{-2}
3×10^{-2}
2×10^{-2}
10^{-2}
graph-tool
igraph
library
Random graphs, m=4n
Transitivity runtime [s]
10^{-1}
10^{-2}
10^{-3}
10^{-4}
10^{-5}
graph-tool
igraph
10^2
10^4
10^6
nodes
Random graphs, m=4n
Construction runtime [s]
10^2
10^1
10^0
10^{-1}
10^{-2}
10^{-3}
10^{-4}
graph-tool
igraph
10^2
10^4
10^6
nodes
