# Supplementary material for: igraph 1.0 enables fast and robust network analysis across programming languages
Source: PLoS One. 2026 Aug 4;21(8):e0355108. doi: 10.1371/journal.pone.0355108 (PMC13436765; doi:10.1371/journal.pone.0355108)
Supplement: Supplementary file 5 [file pone.0355108.s005.pdf]

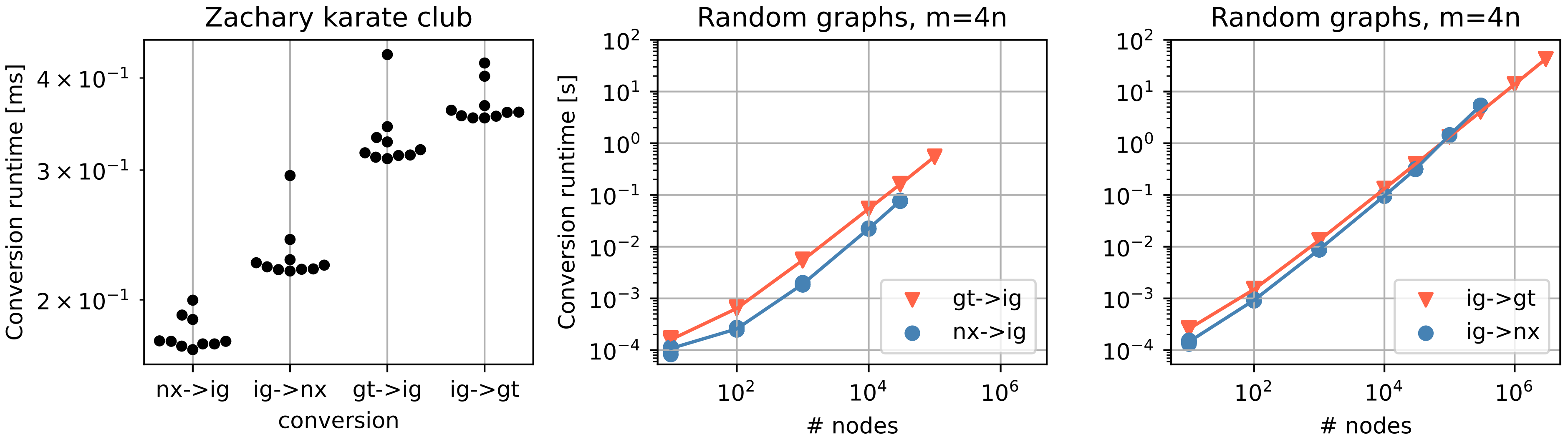

Zachary karate club
Conversion runtime [ms]
4×10^{-1}
3×10^{-1}
2×10^{-1}
nx->ig
ig->nx
gt->ig
ig->gt
conversion
Random graphs, m=4n
Conversion runtime [s]
10^{2}
10^{1}
10^{0}
10^{-1}
10^{-2}
10^{-3}
10^{-4}
gt->ig
nx->ig
10^{2}
10^{4}
10^{6}
nodes
Random graphs, m=4n
10^{2}
10^{1}
10^{0}
10^{-1}
10^{-2}
10^{-3}
10^{-4}
ig->gt
ig->nx
10^{2}
10^{4}
10^{6}
nodes
