# Supplementary material for: igraph 1.0 enables fast and robust network analysis across programming languages
Source: PLoS One. 2026 Aug 4;21(8):e0355108. doi: 10.1371/journal.pone.0355108 (PMC13436765; doi:10.1371/journal.pone.0355108)
Supplement: Supplementary file 6 [file pone.0355108.s007.pdf]

Authors' Response to Reviews of

igraph 1.0 enables fast and robust network analysis across programming languages

Antonov et al.
PLOS ONE,

RC: ***Reviewers' Comment***, AR: Authors' Response

Reviewer #1

AR: We kindly thank the reviewer for their encouraging comments and recommendation to accept for publication.

Reviewer #2

AR: Thank you for your insightful comments.

RC: ***Major concerns:***

1. Performance comparisons are limited

RC: ***The benchmarks compare igraph only against NetworkX. The introduction itself acknowledges that graph-tool is faster than NetworkX, yet graph-tool is absent from all performance comparisons. The authors should either include comparisons with graph-tool or provide a clear justification for its exclusion. Graph-tool, SNAP, and Boost Graph are discussed in the Introduction but excluded from the main performance comparisons.***

AR: We now include a few benchmarks against graph-tool and against scikit-network as requested by reviewer 3 (Supplementary Figure 4) and have updated the methods accordingly.

More generally, we appreciate the request to benchmark igraph across the landscape of network analysis tools. However, a robust benchmarking study would incur significant complexity including: insuring that the same algorithm is used across tools, evaluating which approximations are used by which tool and how they affect the results, consistent compiler flags for compiled languages, deep knowledge of all tools to ensure the most efficient approach is used for each one, and so on. That kind of study would be really interesting and is beyond the scope of the present manuscript. To facilitate such comparison in a future study, we now also mention NetworKit and rustworkx in the introduction.

2. Billion edge performance claims are incomplete

RC: ***The paper demonstrates that igraph can construct graphs with billions of edges, which is impressive. However, it does not show any analytical stuffs like centrality measures, or community detection perform well at this scale. Construction and analysis are different tasks, and the latter is what most users actually need. The authors should either provide benchmarks for analytical operations at large scale or moderate their claims accordingly.***

AR: Thank you for raising this point. We have now added demonstrations of graph analysis, in addition to construction, for graphs of the order of 1 billion edges in Figure 1A (bottom row). Each runtime was measured 10 times and excludes the time needed to construct the graph and store it in RAM.

3. Benchmarking methodology needs strengthening

RC: ***Each graph size was tested only twice. While the performance claims are carefully worded, more repetitions with reported variance would make the results more convincing and allow readers to assess the reliability of the reported speedups.***

AR: This is a fair point. We have now repeated these comparisons for 10 additional times at each graph size and found very similar results. Figures and methods were updated accordingly.

4. Diversity and community section lacks quantitative evidence

RC: ***The manuscript discusses efforts to increase diversity and community participation, including collaboration with Women in Network Science. However, no data is provided on the impact of these initiatives. For example, has the proportion of contributions from underrepresented groups changed? Quantitative evidence would strengthen this section considerably.***

AR: We appreciate this request for quantitative evidence of a shift in the user and contributor base as a result of our diversity efforts. While we do not track the identities of individual users and contributors and therefore cannot speak directly to shifting demographics, we know for certain that at least 25 new women users were engaged through targeted workshops, and we have implemented new governance strategies that are associated with increase recruitment and retention of diverse users and contributors. We have adjusted the paragraph on diversity to reflect this evidence. We now also cite a preprint by some of us that details that study in more depth (Lange et al. arXiv 2025, `https://doi.org/10.48550/arXiv.2503.13405`).

5. Layouts

RC: ***The UMAP layout shown in Figure 2 is presented as an example of igraph's visualisation capability. However, the figure would be more informative if the UMAP layout were shown alongside other commonly used layout algorithms.***

AR: This is a great suggestion. We have now updated Figure 2 with a direct – if anecdotal – comparison between Fruchterman-Reingold (force or energy-based) and UMAP for graphs of various sizes. Except for the largest graph, for which FR could not be run, the same exact graph is used as basis for both algorithms. In addition to showcasing the visualisation capabilities of *igraph*, this example shows how FR works well even for small graphs while UMAP tends to lose resolution, while large graphs cannot be computed by FR due to its worse scalability.

6. Interoperability

RC: ***The interoperability example in Figure 3 demonstrates that graphs can be converted between igraph and networkx. However, for users working with large graphs, the practical feasibility of this conversion depends on its speed and memory cost. The authors should provide information on conversion speed and memory usage across a range of graph sizes, so users can make informed decisions about whether conversion is practical for their use case.***

AR: This is a fair comment. The answer does depend on what one means by "large" graph. The memory

duplication involved in this kind of conversion makes it unsuitable for graphs that occupy almost the entire RAM available on the system. Storing to file, which igraph can also do with multiple supported formats, is the preferred option in those cases. Performance in terms of runtime does depend on the individual file format. The in-memory conversion facility is meant to cover the rest of the size spectrum, i.e. graphs (including large but not entire RAM-sized / larger-than-RAM ones) that require analytical tools scattered across *igraph*, *networkx*, and *graph-tool*. We have now added runtime measurements of conversions between *igraph* and the other two tools for a number of examples and added the results in Supplementary Figure 5. We also found that for some graph constructors, it is faster to construct the graph in *igraph* and then convert it in-memory to the library of choice than to use native construction routines in the external library itself.

Reviewer #3

RC: ***The paper presents igraph 1.0, a general-purpose graph analysis library. igraph has been in development since the last twenty years and the current release in the paper is capable of processing graphs with billions of edges, offers interactive visualization capabilities and integrates seamlessly with notebook environments.***

The paper is well-written and insightful to read. As the authors mentioned, the development of fast and consistent graph analysis libraries across programming languages is attracting increasing research attention and remains highly important particularly given the growing scale and complexity of networks enabled by modern AI and large language models (e.g., in domains such as social network analysis and bioinformatics). The framework's generic design facilitates deeper integration with machine learning ecosystems, particularly as graph neural networks have become increasingly prominent for tasks such as node classification, link prediction and other related graph-level prediction problems. In this context, igraph is a valuable component within modern graph-based analysis experiments.

AR: Thank you for your valuable assessment and for your time.

RC: ***The paper has the following issues (no major comments):***

1. For the NetworkX comparisons, what version was tested? Have comparisons been run against NetworkX 3.0+?

AR: Yes, the comparisons were run against *networkx* 3.0+. We have now computed 10 more repeats of all tests while logging the networkx version (3.6.1) found no visible difference from previous results. We updated the figures and methods accordingly.

RC: ***2. It would be valuable for the authors to report memory usage benchmarks in addition to runtime results for billion-edge graphs.***

AR: We now assess max memory usage during computation of global transitivity and Leiden clustering for graphs of various sizes (Supplementary Figure 2), which mirror runtime assessments for these two routines in the new Figure 1B.

RC: ***3. The authors should also consider comparing igraph with scikit-network, which provides optimized implementations for large-scale graph mining tasks (e.g., clustering, ranking and diffusion) that are not always efficiently supported in standard graph analysis libraries.***

AR: We have now added a comparison with *scikit-network* in Supplementary Figure 4 and updated the methods. As noted in response to a similar query by reviewer 2, this manuscript is not aiming to provide accurate benchmarks across network libraries for various kinds of user needs – although such a study would be really

interesting. Therefore, although we attempted our best at providing a fair assessment of runtime performance during manuscript revision, the results should be taken with a grain of salt. We have also added a citation to scikit-network's paper in the Discussion section about machine learning on graphs.

RC: ***The study represents an important contribution to advancing the development of efficient and consistent graph analysis tools across programming languages.***

AR: Thank you for your comment.
