# Supplementary material for: igraph 1.0 enables fast and robust network analysis across programming languages
Source: PLoS One. 2026 Aug 4;21(8):e0355108. doi: 10.1371/journal.pone.0355108 (PMC13436765; doi:10.1371/journal.pone.0355108)
Supplement: Peer review history

# Peer review: Decision Letter 0

**DOI:** 10.1371/journal.pone.0355108.r001

**Authors:** Amit Nanavati

3 Jun 2026

PONE-D-26-21907

igraph 1.0 enables fast and robust network analysis across programming languages

PLOS One

Dear Dr. Zanini,

Thank you for submitting your manuscript to PLOS ONE. After careful consideration, we feel that it has merit but does not fully meet PLOS ONE’s publication criteria as it currently stands. Therefore, we invite you to submit a revised version of the manuscript that addresses the points raised during the review process.

- **Kindly address the concerns raised by Reviewer#2 and Reviewer#3.**

Please submit your revised manuscript by Jul 18 2026 11:59PM. If you will need more time than this to complete your revisions, please reply to this message or contact the journal office at plosone@plos.org. When you're ready to submit your revision, log on to https://www.editorialmanager.com/pone/ and select the 'Submissions Needing Revision' folder to locate your manuscript file.

Please include the following items when submitting your revised manuscript:

- A letter that responds to each point raised by the academic editor and reviewer(s). You should upload this letter as a separate file labeled 'Response to Reviewers'.
- A marked-up copy of your manuscript that highlights changes made to the original version. You should upload this as a separate file labeled 'Revised Manuscript with Track Changes'.
- An unmarked version of your revised paper without tracked changes. You should upload this as a separate file labeled 'Manuscript'.

If you would like to make changes to your financial disclosure, please include your updated statement in your cover letter. Guidelines for resubmitting your figure files are available below the reviewer comments at the end of this letter.

If applicable, we recommend that you deposit your laboratory protocols in protocols.io to enhance the reproducibility of your results. Protocols.io assigns your protocol its own identifier (DOI) so that it can be cited independently in the future. For instructions see: https://journals.plos.org/plosone/s/submission-guidelines#loc-laboratory-protocols. Additionally, PLOS ONE offers an option for publishing peer-reviewed Lab Protocol articles, which describe protocols hosted on protocols.io. Read more information on sharing protocols at https://plos.org/protocols?utm_medium=editorial-email&utm_source=authorletters&utm_campaign=protocols.

As the corresponding author, your ORCID iD is verified in the submission system and will appear in the published article. PLOS supports the use of ORCID, and we encourage all coauthors to register for an ORCID iD and use it as well. Please encourage your coauthors to verify their ORCID iD within the submission system before final acceptance, as unverified ORCID iDs will not appear in the published article. *Only*  the individual author can complete the verification step; PLOS staff *cannot*  verify ORCID iDs on behalf of authors.

We look forward to receiving your revised manuscript.

Kind regards,

Amit Nanavati

Academic Editor

PLOS One

**Journal Requirements:**

1. When submitting your revision, we need you to address these additional requirements.

Please ensure that your manuscript meets PLOS ONE's style requirements, including those for file naming. The PLOS ONE style templates can be found at

https://journals.plos.org/plosone/s/file?id=wjVg/PLOSOne_formatting_sample_main_body.pdf and

https://journals.plos.org/plosone/s/file?id=ba62/PLOSOne_formatting_sample_title_authors_affiliations.pdf

2. Please update your submission to use the PLOS LaTeX template. The template and more information on our requirements for LaTeX submissions can be found at http://journals.plos.org/plosone/s/latex.

3. Please note that PLOS One has specific guidelines on code sharing for submissions in which author-generated code underpins the findings in the manuscript. In these cases, we expect all author-generated code to be made available without restrictions upon publication of the work. Please review our guidelines at https://journals.plos.org/plosone/s/materials-and-software-sharing#loc-sharing-code and ensure that your code is shared in a way that follows best practice and facilitates reproducibility and reuse.

4. We note that the grant information you provided in the ‘Funding Information’ and ‘Financial Disclosure’ sections do not match.

When you resubmit, please ensure that you provide the correct grant numbers for the awards you received for your study in the ‘Funding Information’ section.

5. Thank you for stating in your Funding Statement:

Development of igraph and its interfaces is supported by two Chan Zuckerberg Essential Open Source Software (EOSS) grants (N. EOSS2-0000000067 and EOSS4-0000000179) and a Chan Zuckerberg Diversity grant (N. EOSS-DI-0000000021).

Please provide an amended statement that declares *all* the funding or sources of support (whether external or internal to your organization) received during this study, as detailed online in our guide for authors at http://journals.plos.org/plosone/s/submit-now.  Please also include the statement “There was no additional external funding received for this study.” in your updated Funding Statement.

Please include your amended Funding Statement within your cover letter. We will change the online submission form on your behalf.

6. Thank you for stating the following financial disclosure:

Development of igraph and its interfaces is supported by two Chan Zuckerberg Essential Open Source Software (EOSS) grants (N. EOSS2-0000000067 and EOSS4-0000000179) and a Chan Zuckerberg Diversity grant (N. EOSS-DI-0000000021).

Please state what role the funders took in the study.  If the funders had no role, please state: "The funders had no role in study design, data collection and analysis, decision to publish, or preparation of the manuscript."

If this statement is not correct you must amend it as needed.

Please include this amended Role of Funder statement in your cover letter; we will change the online submission form on your behalf.

7. In the online submission form, you indicated that [The datasets used and/or analysed during the current study available from the corresponding author on reasonable request. The code is available at https://igraph.org.].

All PLOS journals now require all data underlying the findings described in their manuscript to be freely available to other researchers, either a. In a public repository, b. Within the manuscript itself, or c. Uploaded as supplementary information.

This policy applies to all data except where public deposition would breach compliance with the protocol approved by your research ethics board. If your data cannot be made publicly available for ethical or legal reasons (e.g., public availability would compromise patient privacy), please explain your reasons on resubmission and your exemption request will be escalated for approval.

8. Please upload a new copy of Figures 1, 2 and 3, as the detail is not clear. Please follow the link for more information:  https://journals.plos.org/plosone/s/figures

9. We note you have included a Supplementary figures and table to which you do not refer in the text of your manuscript. Please ensure that you refer to Supplementary figures 1, 2 and table 1 in your text; if accepted, production will need this reference to link the reader to the Supplementary figures and table.

10. If the reviewer comments include a recommendation to cite specific previously published works, please review and evaluate these publications to determine whether they are relevant and should be cited. There is no requirement to cite these works unless the editor has indicated otherwise.

11. Please review your reference list to ensure that it is complete and correct. If you have cited papers that have been retracted, please include the rationale for doing so in the manuscript text, or remove these references and replace them with relevant current references. Any changes to the reference list should be mentioned in the rebuttal letter that accompanies your revised manuscript. If you need to cite a retracted article, indicate the article’s retracted status in the References list and also include a citation and full reference for the retraction notice.

**Additional Editor Comments:**

Thank you very much for your submission!

I request you to address the concerns of Reviewers 2 and 3 and resubmit after revising.

Thank you very much!

[Note: HTML markup is below. Please do not edit.]

Reviewers' comments:

Reviewer's Responses to Questions

**Comments to the Author**

1. Is the manuscript technically sound, and do the data support the conclusions?

The manuscript must describe a technically sound piece of scientific research with data that supports the conclusions. Experiments must have been conducted rigorously, with appropriate controls, replication, and sample sizes. The conclusions must be drawn appropriately based on the data presented.

Reviewer #1: Yes

Reviewer #2: No

Reviewer #3: Yes

**********

2. Has the statistical analysis been performed appropriately and rigorously?

Reviewer #1: Yes

Reviewer #2: Yes

Reviewer #3: N/A

**********

3. Have the authors made all data underlying the findings in their manuscript fully available?

The PLOS Data policy requires authors to make all data underlying the findings described in their manuscript fully available without restriction, with rare exception (please refer to the Data Availability Statement in the manuscript PDF file). The data should be provided as part of the manuscript or its supporting information, or deposited to a public repository. For example, in addition to summary statistics, the data points behind means, medians and variance measures should be available. If there are restrictions on publicly sharing data—e.g. participant privacy or use of data from a third party—those must be specified.

Reviewer #1: Yes

Reviewer #2: No

Reviewer #3: Yes

**********

4. Is the manuscript presented in an intelligible fashion and written in standard English?

PLOS ONE does not copyedit accepted manuscripts, so the language in submitted articles must be clear, correct, and unambiguous. Any typographical or grammatical errors should be corrected at revision, so please note any specific errors here.

Reviewer #1: Yes

Reviewer #2: Yes

Reviewer #3: Yes

**********

5. Review Comments to the Author

Please use the space provided to explain your answers to the questions above. You may also include additional comments for the author, including concerns about dual publication, research ethics, or publication ethics. (Please upload your review as an attachment if it exceeds 20,000 characters)

**Reviewer #1:**  My reasons for acceptance:

1. igraph has established itself over two decades as one of the most relied-upon libraries for graph and network analysis.

2. C core for performance: the core being written in C, igraph achieves much faster performance than pure python based implementations like networkx for graph analysis and visualisation.

3. Cross-language accessibility and interoperability: igraph's support for Python, R, and Mathematica through a shared C core is a valuable design choice. There is also compatibility with networkx.

4. Open science and open source: The transparent development model reflects best practices in open science..

**Reviewer #2:**  Major concerns:

1. Performance comparisons are limited

The benchmarks compare igraph only against NetworkX. The introduction itself acknowledges that graph-tool is faster than NetworkX, yet graph-tool is absent from all performance comparisons. The authors should either include comparisons with graph-tool or provide a clear justification for its exclusion. Graph-tool, SNAP, and Boost Graph are discussed in the Introduction but excluded from the main performance comparisons.

2. Billion edge performance claims are incomplete

The paper demonstrates that igraph can construct graphs with billions of edges, which is impressive. However, it does not show any analytical stuffs like centrality measures, or community detection perform well at this scale. Construction and analysis are different tasks, and the latter is what most users actually need. The authors should either provide benchmarks for analytical operations at large scale or moderate their claims accordingly.

3. Benchmarking methodology needs strengthening

Each graph size was tested only twice. While the performance claims are carefully worded, more repetitions with reported variance would make the results more convincing and allow readers to assess the reliability of the reported speedups.

4. Diversity and community section lacks quantitative evidence

The manuscript discusses efforts to increase diversity and community participation, including collaboration with Women in Network Science. However, no data is provided on the impact of these initiatives. For example, has the proportion of contributions from underrepresented groups changed? Quantitative evidence would strengthen this section considerably.

5.The UMAP layout shown in Figure 2 is presented as an example of igraph's visualisation capability. However, the figure would be more informative if the UMAP layout were shown alongside other commonly used layout algorithms.

6.The interoperability example in Figure 3 demonstrates that graphs can be converted between igraph and networkx. However, for users working with large graphs, the practical feasibility of this conversion depends on its speed and memory cost. The authors should provide information on conversion speed and memory usage across a range of graph sizes, so users can make informed decisions about whether conversion is practical for their use case.

**Reviewer #3:**  The paper presents igraph 1.0, a general-purpose graph analysis library. igraph has been in development since the last twenty years and the current release in the paper is capable of processing graphs with billions of edges, offers interactive visualization capabilities and integrates seamlessly with notebook environments.

The paper is well-written and insightful to read. As the authors mentioned, the development of fast and consistent graph analysis libraries across programming languages is attracting increasing research attention and remains highly important particularly given the growing scale and complexity of networks enabled by modern AI and large language models (e.g., in domains such as social network analysis and bioinformatics). The framework's generic design facilitates deeper integration with machine learning ecosystems, particularly as graph neural networks have become increasingly prominent for tasks such as node classification, link prediction and other related graph-level prediction problems. In this context, igraph is a valuable component within modern graph-based analysis experiments.

The paper has the following issues (no major comments):

1. For the NetworkX comparisons, what version was tested? Have comparisons been run against NetworkX 3.0+?

2. It would be valuable for the authors to report memory usage benchmarks in addition to runtime results for billion-edge graphs.

3. The authors should also consider comparing igraph with scikit-network, which provides optimized implementations for large-scale graph mining tasks (e.g., clustering, ranking and diffusion) that are not always efficiently supported in standard graph analysis libraries.

The study represents an important contribution to advancing the development of efficient and consistent graph analysis tools across programming languages.

**********

6. PLOS authors have the option to publish the peer review history of their article (what does this mean?). If published, this will include your full peer review and any attached files.

If you choose “no”, your identity will remain anonymous but your review may still be made public.

**Do you want your identity to be public for this peer review?**  For information about this choice, including consent withdrawal, please see our Privacy Policy.

Reviewer #1: No

Reviewer #2: No

Reviewer #3: No

**********

[NOTE: If reviewer comments were submitted as an attachment file, they will be attached to this email and accessible via the submission site. Please log into your account, locate the manuscript record, and check for the action link "View Attachments". If this link does not appear, there are no attachment files.]

To ensure your figures meet our technical requirements, please review our figure guidelines: https://journals.plos.org/plosone/s/figures

You may also use PLOS’s free figure tool, NAAS, to help you prepare publication quality figures: https://journals.plos.org/plosone/s/figures#loc-tools-for-figure-preparation.

NAAS will assess whether your figures meet our technical requirements by comparing each figure against our figure specifications.

# Author comment: Author response to Decision Letter 1

**DOI:** 10.1371/journal.pone.0355108.r002

13 Jul 2026

(See PDF with point-by-point responses to reviewers' comments)

Attachment Submitted filename: responses_to_reviewers.pdf

# Editor report: Decision Letter 1

**DOI:** 10.1371/journal.pone.0355108.r003

**Authors:** Amit Nanavati

17 Jul 2026

igraph 1.0 enables fast and robust network analysis across programming languages

PONE-D-26-21907R1

Dear Dr. Zanini,

We’re pleased to inform you that your manuscript has been judged scientifically suitable for publication and will be formally accepted for publication once it meets all outstanding technical requirements.

Within one week, you’ll receive an e-mail detailing the required amendments. When these have been addressed, you’ll receive a formal acceptance letter and your manuscript will be scheduled for publication.

An invoice will be generated when your article is formally accepted. Please note, if your institution has a publishing partnership with PLOS and your article meets the relevant criteria, all or part of your publication costs will be covered. Please make sure your user information is up-to-date by logging into Editorial Manager at Editorial Manager® and clicking the ‘Update My Information' link at the top of the page. For questions related to billing, please contact billing support.

If your institution or institutions have a press office, please notify them about your upcoming paper to help maximize its impact. If they’ll be preparing press materials, please inform our press team as soon as possible -- no later than 48 hours after receiving the formal acceptance. Your manuscript will remain under strict press embargo until 2 pm Eastern Time on the date of publication. For more information, please contact onepress@plos.org.

Kind regards,

Amit Nanavati

Academic Editor

PLOS One

Additional Editor Comments (optional):

Reviewers' comments:

# Editor report: Acceptance letter

**DOI:** 10.1371/journal.pone.0355108.r004

**Authors:** Amit Nanavati

PONE-D-26-21907R1

PLOS One

Dear Dr. Zanini,

I'm pleased to inform you that your manuscript has been deemed suitable for publication in PLOS One. Congratulations! Your manuscript is now being handed over to our production team.

At this stage, our production department will prepare your paper for publication. This includes ensuring the following:

* All references, tables, and figures are properly cited

* All relevant supporting information is included in the manuscript submission,

* There are no issues that prevent the paper from being properly typeset

You will receive further instructions from the production team, including instructions on how to review your proof when it is ready. Please keep in mind that we are working through a large volume of accepted articles, so please give us a few days to review your paper and let you know the next and final steps.

Lastly, if your institution or institutions have a press office, please let them know about your upcoming paper now to help maximize its impact. If they'll be preparing press materials, please inform our press team within the next 48 hours. Your manuscript will remain under strict press embargo until 2 pm Eastern Time on the date of publication. For more information, please contact onepress@plos.org.

You will receive an invoice from PLOS for your publication fee after your manuscript has reached the completed accept phase. If you receive an email requesting payment before acceptance or for any other service, this may be a phishing scheme. Learn how to identify phishing emails and protect your accounts at https://explore.plos.org/phishing.

If we can help with anything else, please email us at customercare@plos.org.

Thank you for submitting your work to PLOS ONE and supporting open access.

Kind regards,

PLOS ONE Editorial Office Staff

on behalf of

Prof. Amit Nanavati

Academic Editor

PLOS One
